# Human Papillomavirus Infection as a Rare Etiological Factor in Moderate Squamous Dysplasia of the Trachea

**DOI:** 10.3390/diagnostics15222868

**Published:** 2025-11-12

**Authors:** Dana-Maria Avasilcăi, Mihai Alexandru Arghir, Ancuța-Alina Constantin

**Affiliations:** 1 Department of Cardio-Thoracic Pathology, “Carol Davila” University of Medicine and Pharmacy, 050474 Bucharest, Romania; dana-maria.avasilcai@drd.umfcd.ro (D.-M.A.); mihai-alexandru.arghir0125@rez.umfcd.ro (M.A.A.); 2 Institute of Pneumology “Marius Nasta”, 050159 Bucharest, Romania

**Keywords:** HPV subtype 45, squamous dysplasia, trachea

## Abstract

We report the case of a 42-year-old, non-smoking male admitted with right upper-lobe pneumonia. Chest computed tomography (CT) demonstrated findings consistent with an infectious process. For further evaluation, serial bronchoscopies with biopsy sampling were performed. Histopathological examination revealed moderate squamous dysplasia of the tracheal epithelium, and subsequent immunohistochemical testing detected human papillomavirus (HPV) genotype 45. This case underscores the value of integrating imaging, endoscopic assessment, and molecular diagnostic techniques in the evaluation of atypical pulmonary lesions and highlights the potential role of HPV infection in airway epithelial dysplastic changes.

**Figure 1:**
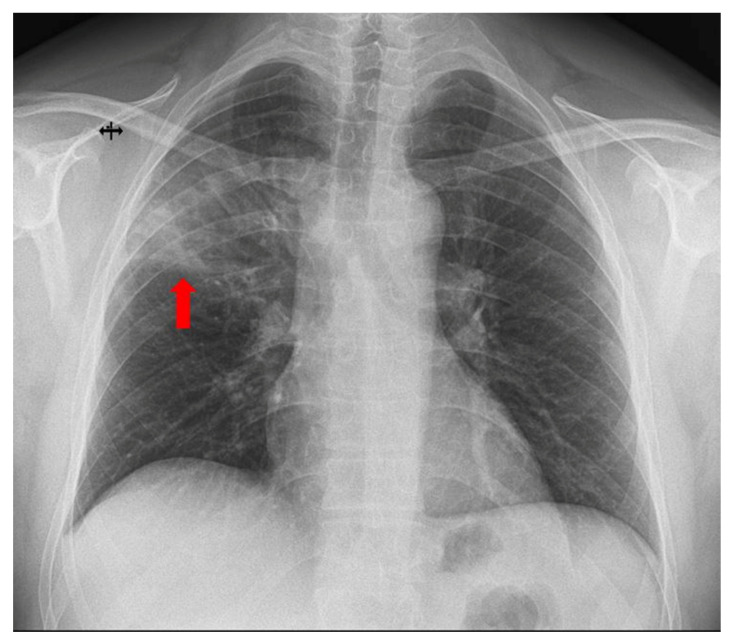
A 42-year-old non-smoking male electrician with occupational exposure to respiratory irritants presented to the emergency department with a 24 h history of fever (38.7 °C), non-productive, irritating cough, and diffuse post-tussive chest pain. His past medical history included SARS-CoV-2 infection four years earlier and mixed dyslipidemia controlled with statins and fenofibrate. On admission, he was febrile (38.7 °C), normotensive, and maintained normal oxygen saturation. Lung auscultation revealed bilateral vesicular breath sounds without additional adventitious sounds. Laboratory evaluation demonstrated an inflammatory response (elevated C-reactive protein—108 mg/L (0–5 mg/L) and procalcitonin—0.17 ng/mL (0–0.05 ng/mL)), with normal value of leukocytes (WBC—9.99 × 10^3^/µL, neutrophils—6.86 × 103/µL, lymphocytes—1.82 × 10^3^/µL), and hypertriglyceridemia. Chest X-ray revealed a subpleural, pseudo-triangular opacity of inhomogeneous density in the upper third of the right lung field (red arrow). For further evaluation of the radiographic abnormality and pulmonary function, spirometry with bronchodilator testing and thoracic CT were recommended.

**Figure 2:**
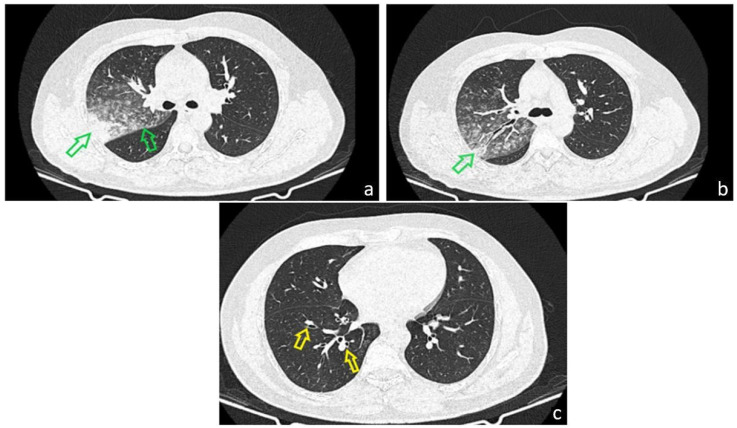
A Chest CT demonstrated solid and subsolid pseudonodular and micronodular infiltrates in the right upper lobe (RUL), showing a tendency to confluence ((**a**), green arrows) and areas with a “tree-in-bud” appearance ((**b**), green arrows) on a background of ground-glass opacity. These findings were initially interpreted as consistent with an infectious process, although serial sputum cultures yielded no identifiable bacterial pathogen. Based on the clinical and imaging profile, a diagnosis of right upper-lobe pneumonia was made, and empirical ceftriaxone therapy was initiated, resulting in resolution of fever, remission of the dry irritative cough, and normalization of inflammatory markers. The CT scan also revealed cylindrical bronchiectasis with wall thickening in the right lower lobe ((**c**), yellow arrow). Pulmonary function testing showed moderate restrictive ventilatory dysfunction (forced vital capacity 66% of predicted) without significant bronchodilator response, and normal DLCO. Given the inconclusive microbiological results, a fiberoptic bronchoscopy was performed to further asses the airway mucosa and rule out endobronchial lesions.

**Figure 3:**
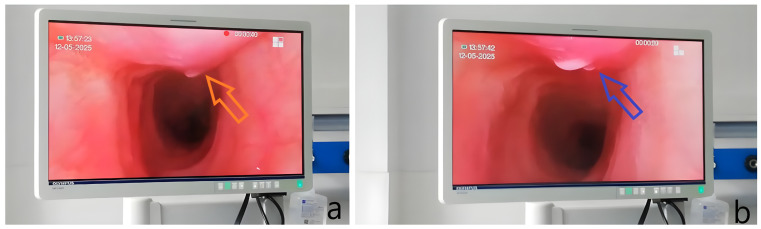
Fiberoptic bronchoscopy revealed diffuse tracheal mucosal changes, extending from the cricoid cartilage: the mucosa appeared circumferentially congested and moderately hypertrophic ((**a**), orange arrow), with whitish deposits on the posterior ((**b**), blue arrow) and, to a lesser extent, lateral walls. These lesions extended into both main bronchi and gradually normalized at the segmental bronchial level. Bronchoalveolar lavage from the RUL bronchus contained numerous neutrophils and macrophages, abundant mucus, and no microbial flora; Ziehl–Neelsen staining was negative for acid-fast bacilli.

**Figure 4:**
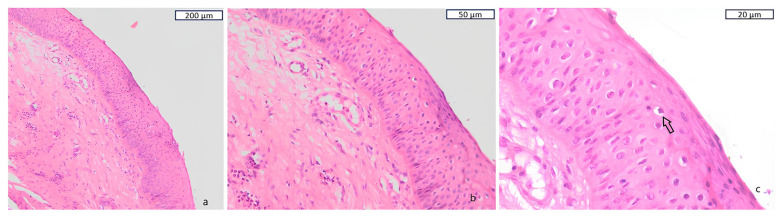
Given the suspicious appearance of the tracheal mucosa, four biopsy specimens were obtained from the posterior wall of the proximal third of the trachea. The initial histopathological examination revealed eight sub-millimetric fragments of superficial bronchial mucosa, focally lined by squamous-metaplastic epithelium. Within the mucosal stroma, isolated intracapsular cellular emboli composed of atypical epithelial elements suspicious for malignancy were identified. Because the specimen was of suboptimal cellularity, a repeat bronchoscopy with additional tissue sampling was performed one week later. The second biopsy demonstrated squamous metaplastic epithelium with areas of moderate to severe dysplasia (**a**), in which two-thirds or nearly the entire epithelial thickness was occupied by cells resembling those of the basal layer (**b**). Additionally, cells resembling koilocytes were observed, characterized by hyperchromatic nuclei with irregular, raisin-like contours and a clear perinuclear halo ((**c**), black arrow). The presence of these cells in the examined specimens raised suspicion for Human Papilloma Virus infection. To further characterize this nearly incidental tracheal lesion, immunohistochemical testing was performed at a reference laboratory and detected HPV DNA, subtype 45, a genotype associated with high oncogenic potential.

**Figure 5:**
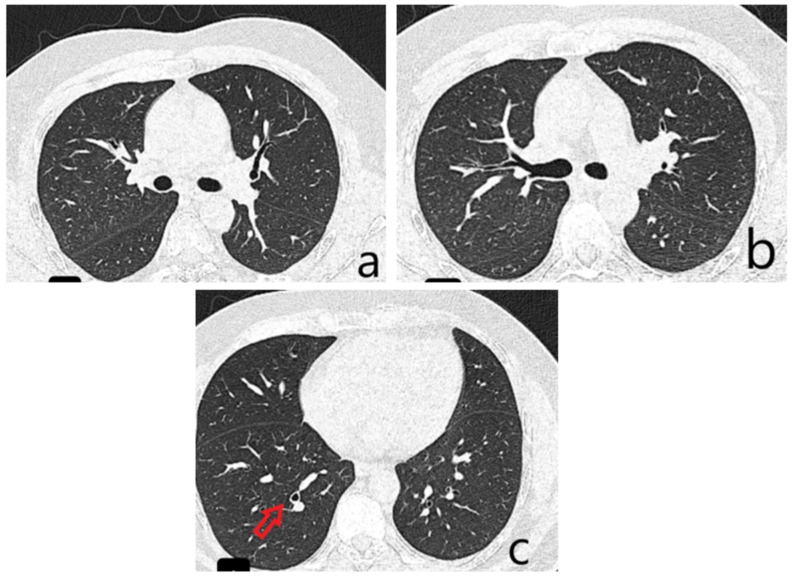
The patient underwent bronchoscopic re-evaluation one month after the initial diagnosis. He was in good general condition, without respiratory symptoms. Follow-up CT imaging demonstrated a favorable evolution, with regression of the pulmonary lesions of infectious appearance (**a**,**b**), while persistent cylindrical bronchiectasis was noted in the lower lobes ((**c**), red arrow). Pulmonary function testing showed improvement in forced vital capacity (FVC) from 66% to 78% of the predicted value, approaching the lower limit of normal. Follow-up fiberoptic bronchoscopy revealed a larynx with preserved dynamics and a mild, diffuse, bilateral congestive appearance. No proliferative elements or active mucosal lesions were detected. A subtle broadening of the bronchial spurs was noted at the intersegmental level of the right upper lobe; otherwise, the mucosa appeared normal. The previously described tracheal lesion was no longer evident, which may reflect a transient immunosuppressive state during pneumonia, leading to reactivation of a latent HPV infection that subsequently regressed as the pulmonary infection resolved. The patient remains under pneumology follow-up, with a scheduled bronchoscopic re-evaluation in three months, given the prior histopathological findings and the immunohistochemical detection of HPV DNA genotype 45. Additionally, the patient was referred to the Gynecology Department for assessment of HPV vaccination eligibility. Although Tracheal tumors represent a diagnostic challenge for clinicians because their symptoms are typically nonspecific and often appear late, with airway obstruction being the most serious and potentially life-threatening manifestation. Most tracheal tumors are malignant, usually arising from direct invasion by adjacent malignancies or via hematogenous dissemination. The often delayed diagnosis significantly narrows the window for potentially curative treatment, such as surgical resection, and contributes to the poor overall 5-year survival rate of approximately 27% [1]. Curative surgery is further limited by the anatomical constraints of the trachea and the extent of tumor invasion. As a result, alternative therapeutic modalities—including chemotherapy, radiotherapy, and advanced bronchoscopic interventions—are more frequently employed for palliation and extension of survival, though none offer a definitive cure. Primary tracheal cancer is rare. In adults, about 90% of primary tracheal neoplasms are malignant, with squamous cell carcinoma and adenoid cystic carcinoma together accounting for over 60% of cases [1]. A wide spectrum of other primary malignant tumors may also arise from the trachea, comprising roughly one-third of cases (≈38.9%) [1]. Reported histologic subtypes include undifferentiated carcinoma, small-cell carcinoma, adenocarcinoma, large-cell carcinoma, melanoma, lymphoma, carcinoid tumors (typical and atypical), and sarcoma. Given the rarity of these tumors, no prospective studies have been conducted; thus, current knowledge about their clinical behavior and optimal management relies largely on case reports and small case series. Risk factors for malignant tracheal tumors differ by histologic type. For squamous cell carcinoma, the strongest associations include tobacco use, alcohol consumption, and a higher prevalence in males. In contrast, adenoid cystic carcinoma shows a more balanced sex distribution and occurs more frequently in non-smokers [1,2]. Dysplasia represents a spectrum of architectural and cytologic abnormalities within the squamous mucosal epithelium and is associated with an increased risk of malignant transformation. Beyond the well-established risk factors of tobacco and alcohol use, gastroesophageal reflux disease has also been implicated, whereas transcriptionally active human papillomavirus (HPV) is generally regarded as playing only a minor role in the pathogenesis of airway dysplasia [3]. This underscores the distinctiveness of our case, in which HPV DNA subtype 45 was identified on immunohistochemical testing of the patient’s tracheal biopsy. We hypothesize that a transient immune imbalance related to pneumonia may have triggered reactivation of a latent HPV infection, thereby contributing to the development of moderate squamous dysplasia of the trachea. This emphasizes the need for clinicians to maintain a broad differential diagnosis and to consider less common etiological factors when evaluating dysplastic airway lesions. Human papillomaviruses (HPVs) comprise over 100 types, with 12 recognized as Group 1 carcinogens—“carcinogenic to humans”—by the International Agency for Research on Cancer (IARC) [4]. Most HPV infections are asymptomatic and spontaneously cleared by the host immune system; however, a subset persists and may, in rare instances, progress to malignancy. Emerging evidence suggests that viral genotype and intra-type genetic variation within high-risk HPVs influence both viral persistence and clinical outcomes. HPV45, a high-risk genotype first identified in 1987 in a woman with recurrent cervical lesions in the United States, belongs to the alpha-7 phylogenetic species, together with HPV18. Like HPV18, HPV45 is more frequently detected in cervical adenocarcinomas than in squamous cell carcinomas. Globally, HPV45 is present in about 5% of cervical cancers, with regional prevalence ranging from 3% in Eastern Asia to 9% in Africa. Based on its enrichment in cervical cancers compared with cytologically normal women, HPV45 is ranked as the third most carcinogenic HPV type, following HPV16 and HPV18 [4]. Genetic variants of HPV45 are currently classified into two major lineages (A and B) and five sublineages (A1, A2, A3, B1, and B2). Lineages differ by approximately 1.0% in whole-genome sequence, while sublineages exhibit 0.5–0.9% nucleotide divergence. Unlike other high-risk HPV types such as HPV16, the relationship between HPV45 genetic variants and cervical cancer risk has not been systematically investigated [4]. Although primary tracheal neoplasms are rare, several HPV-associated tracheal malignancies have been reported. Imai et al. described a 75-year-old man presenting with cough and dyspnea. Chest CT revealed tracheal narrowing caused by an intraluminal mass. Histopathology confirmed papillary squamous cell carcinoma with p16 overexpression, and HPV18 DNA was detected by multiplex PCR. At the 12-month follow-up after adjuvant radiotherapy, the patient showed no local recurrence or distant metastasis [5]. Liu et al. reported a 38-year-old woman with a 16-month history of hemoptysis, chest pain, and intermittent fatigue, initially misdiagnosed with tuberculosis based on a positive interferon-γ release assay. Bronchoscopy revealed tracheal wall congestion and edema with a small lesion on the right lateral tracheal wall. Endoscopic biopsy ultimately confirmed squamous cell carcinoma, and metagenomic next-generation sequencing (mNGS) detected HPV16 DNA in the tumor tissue. Testing of her sexual partner demonstrated HPV16 positivity in penile scrapings, supporting the hypothesis of oral-sexual transmission [6]. Suzuki et al. described a 34-year-old man with dyspnea, in whom CT revealed a 2.0 × 1.6 × 1.0 cm polypoid tumor at the tracheal carina with possible extratracheal extension. Urgent bronchoscopic resection was performed both to establish a diagnosis and to prevent impending airway obstruction. Histopathology showed well-differentiated squamous cell carcinoma, and multiplex PCR identified HPV6 DNA in the resected specimen. Following carinal extraction and reconstruction, the patient remained recurrence-free and without anastomotic complications at 1-year follow-up and did not require adjuvant therapy [7]. HPV45 is a recognized high-risk HPV genotype implicated in several cancers, including cervical, anal, and genital malignancies [4]. Although HPV is an established etiologic factor in oropharyngeal cancers, its specific association with laryngeal and tracheal tumors remains insufficiently defined, largely due to the rarity of primary tracheal malignancies and the limited number of reported HPV-related tracheal cases in the literature. This case highlights the critical role of histopathological assessment of tracheal biopsy specimens, complemented by immunohistochemical testing, in identifying underlying etiologic factors such as HPV infection. Early recognition of such uncommon associations may inform patient management, surveillance strategies, and potential preventive measures, including consideration of HPV vaccination in appropriate populations.

## Data Availability

No new data were created or analyzed in this study. Data sharing is not applicable to this article.
